# A comprehensive review of 46 exercise treatment studies in fibromyalgia (1988–2005)

**DOI:** 10.1186/1477-7525-4-67

**Published:** 2006-09-25

**Authors:** Kim Dupree Jones, Dianne Adams, Kerri Winters-Stone, Carol S Burckhardt

**Affiliations:** 1 School of Nursing, Oregon Health & Science University, Portland, Oregon, USA; 2 Division of Arthritis & Rheumatic Diseases, Oregon Health & Science University, Portland, Oregon, USA

## Abstract

The purpose of this review was to: (1) locate all exercise treatment studies of fibromyalgia (FM) patients from 1988 through 2005, (2) present in tabular format the key details of each study and (3) to provide a summary and evaluation of each study for exercise and health outcomes researchers.

Exercise intervention studies in FM were retrieved through Cochrane Collaboration Reviews and key word searches of the medical literature, conference proceedings and bibliographies. Studies were reviewed for inclusion using a standardized process. A table summarizing subject characteristics, exercise mode, timing, duration, frequency, intensity, attrition and outcome variables was developed. Results, conclusions and comments were made for each study. Forty-six exercise treatment studies were found with a total of 3035 subjects. The strongest evidence was in support of aerobic exercise a treatment prescription for fitness and symptom and improvement. In general, the greatest effect and lowest attrition occurred in exercise programs that were of lower intensity than those of higher intensity. Exercise is a crucial part of treatment for people with FM. Increased health and fitness, along with symptom reduction, can be expected with exercise that is of appropriate intensity, self-modified, and symptom-limited. Exercise and health outcomes researchers are encouraged to use the extant literature to develop effective health enhancing programs for people with FM and to target research to as yet understudied FM subpopulations, such as children, men, older adults, ethnic minorities and those with common comorbidities of osteoarthritis and obesity.

## Review

Fibromyalgia (FM) is a pain disorder defined by chronic widespread pain and multiple muscle-tendon junction tender points. Like most chronic illnesses, however, the symptoms of FM extend far beyond the defining criteria. Many patients also report fatigue, disrupted or nonrefreshed sleep, mood disturbances, exercise induced symptom flares and multiple other syndromes (e.g., restless legs, irritable bowel and bladder, and chronic headaches) [1,2]. Physical and emotional health as well as quality of life is often seriously impaired [3,4].

Exercise has been suggested as a treatment for FM since Moldofsky first demonstrated that fit people were less likely to develop FM symptoms when their stages 3 and 4 sleep was intentionally disrupted [5]. The first exercise intervention in FM was published in 1988 and since that time a large number of clinical trials have been reported. In 1999, a meta-analysis established that exercise produced higher effect sizes on physical status, FM symptoms and daily functioning than pharmacological treatment [6]. A recent evidence-based review concluded that cardiovascular exercise is as effective in decreasing pain and FM impact as drugs such as amitriptyline [7].

A number of exercise intervention reviews have been published over the years [8-10]. All of them offer valuable synthesis and critiques based on the authors' expertise. However, they are limited somewhat in comprehensiveness and do not provide descriptions and critiques of each individual study that could be helpful to an exercise or health outcomes researcher who wishes to extend the scope of knowledge in the area.

The purpose of this review paper is to present a comprehensive evidence table of exercise studies with the anticipation that this individual study tabular format and accompanying comments will be useful to exercise and health outcomes researchers seeking to apply their expertise to FM clinical populations.

## Methods

Article titles with their abstracts were accessed through an English language search of Cochrane Collaboration Reviews, MEDLINE, CINAHL, EMBASE, PubMed, Healthstar, Current Contexts, Web of Science, and PsychInfo & Science Citation Indexes. Keyword MeSH terms for initial inclusion were "fibromyalgia" and "exercise" and resulted in 296 'hits'. A further 37 articles and abstracts were found through hand searching of journals, conference proceedings, bibliographies of selected papers and personal contact with key exercise researchers in the field. The first author (KDJ) reviewed all 333 abstracts using standardized criteria developed to determine what type of design the paper reported [11]. After this preliminary step, those that were found to be reviews, case studies, clinical or theoretical papers, and descriptive or correlational cross-sectional studies were excluded. Those that met minimum criteria for an experiemtnal study (i.e. a sample drawn from an FM population, longitudinal design with pre and post measurement of an outcome variable determined *a priori*, and an experimental treatment) were included. In a second step, the first and second authors (KDJ and DA) independently extracted the study design, number of subjects, subject characteristics (age, gender), type of exercise, treatment length, frequency, duration and intensity of the exercise, attrition, and outcome variables from the methods section of the full text articles or from the abstract, if only the abstract was available. Any disagreements were discussed and a consensus obtained between the two raters.

Both randomized controlled trials (RCTs) and uncontrolled trials were included in order to offer the broadest view of the exercise interventions in FM. Trials had to have enrolled FM subjects who met standardized criteria for FM diagnosis that were acceptable at the time the study was done [12,13]. Study interventions had to meet general criteria for some type of physical movement but did not have to contain a physical fitness outcome measure. Thus, low-intensity modalities such as QiGong and T'ai Chi were included. Studies that educated patients regarding how to exercise but did not have any supervised exercise sessions were excluded. However, some of the studies included in this review table had strong educational and cognitive behavioral components, which may have influenced outcomes.

### Results of the evidence review

Results of the review are described and commented upon in Table 1 (see Additional file 1). Studies are listed in chronological order by year. The following paragraphs summarize the findings by each column in the table.

### Subjects

Through December 2005, 3035 subjects participated in an FM exercise study. Of that number, 2888 (2400 women, 73 men, 415 gender not reported) were patients with FM. Control subjects with various chronic diseases other than FM numbered 135 and there were 12 healthy control subjects. The ages of subjects ranged from 18–80 years, with a mean of 49.5 years. Older adults, men, and minority persons were underrepresented and no exercise interventions with children were found.

### Modes of training and control interventions

Most interventions were comprised of the three major modes of exercise (aerobic training, strength training, flexibility) either singly or in combination. Aerobic training included cycling, walking, calisthenics, pool exercise or dance. Thirty-three were land-based. Seven were exclusively water/pool based while the remainder either progressed from water to land based or mixed water and land throughout the intervention. Five studies used only weight training, either machine weights or free weights (hand weights/elastic bands). Progression was determined by changes in number of repetitions (reps), sets or increasing load (e.g., progressive 8–20 reps, 4–6 sets, and load increased by 40–80%) or progression of elastic band tension. Three studies tested flexibility as either the active intervention or the control intervention. Stretches were described as static and progressed by self-limited tension and discomfort or an increase in time from 10–90 seconds per major muscle group. Three studies tested the independent and combined effect of a drug and exercise (amitriptyline and pyridostigmine). Four used movement therapies (e.g. T'ai Chi, QiGong, balneotherapy, thalassotherapy). We acknowledge that other therapies using balneotherapy in FM exist as a modality for treating symptoms, but are not included in this review as they were not combined with exercise.

### Intensity of aerobic training

Aerobic intensity was reported in 14 studies as target heart rate or percent age-predicted maximum heart rate determined by standard equations. No study set work rate based on initial maximal aerobic capacity determined by graded exercise test. Sustained target heart rate goals ranged from 120–150 beats per minute. Percent maximum heart rates were usually progressive and ranged from 40%–80% of age-predicted maximum. Borg's Rating of Perceived Exertion (RPE) scale or the "ability to talk test" was used in two studies. Measuring heart rate was most often accomplished by self-assessed pulse rate or less frequently by heart rate telemetry.

### Frequency of exercise sessions and duration of training

The number of exercise sessions ranged from 1–5 times per week most commonly 2–3 times weekly. Length of class time ranged from 15–180 minutes per session with the average being 60 minutes. The length of the interventions, excluding follow-up, ranged from 4–24 weeks; the median was 12 weeks.

### Attrition and compliance

Attrition in FM subjects ranged from 0–67% (median 20%, mean 21%) while controls ranged from 0–48% (median 8%, mean 14%). Compliance was not calculable in the majority of studies. Some studies analyzed data on intent to treat basis and did not report number of sessions subjects attended. Others stated that "the majority" of subjects completed a certain number of classes or that there was a natural break in the data at a certain number of classes. This is problematic in that the "dose" of the intervention was not generally attainable.

### Outcome measures

The outcome measures in most studies were one or more FM symptoms, measured either on a visual analogue scale, the Fibromyalgia Impact Questionnaire [14] or a health status measure. Fewer also measured fitness markers (strength, flexibility, aerobic capacity). The timing of the measures were pre- vs. post- as compared to multiple time points during the intervention Most failed to explicitly state which outcome was their *a priori *primary dependent variable. None used real-time symptom monitoring with electronic diaries.

### Methodological rigor

Thirty-nine of the studies were randomized, controlled trials with examiners blinded to treatment allocation. The remaining seven were single group (6 studies), or non-randomly assigned multi-group interventions (1 study). Statistical analyses ranged from questionable paired t-tests, uncorrected for multiple comparisons with no stated *a priori *hypothesis and within group changes (paired t-tests, change scores and effect sizes) to appropriate statistical methods including independent group t-test, ANOVA and ANCOVA. Abstracts as opposed to full text articles often had inadequate descriptions of methods and analyses making it difficult to confirm the validity of their stated conclusions.

### Major findings

• Most fitness measures improved in people who could tolerate the intervention (e.g., 1 – RM or isokinetic dynamometry strength, time on treadmill, V0_2 _max or peak, 6 – minute walk, flexibility).

• The exercise interventions in most studies did not meet the current exercise recommendation for health as developed by the Centers for Disease Control and Prevention and the American College of Sports Medicine [15,16] (30 minutes of moderate intensity exercise on most days of the week for health related benefits).

• Those studies that used a higher heart rate or RPE, higher impact movements (e.g., running, jumping) or those where subjects could not self-adjust exercise intensity (e.g. during a flare) suffered the highest attrition rates.

• Subjects attained symptom relief, particularly decreased pain and fatigue as well as improved sleep and mood, with low to moderate intensity exercise of any type. Even very low movement therapies such as QiGong had significant effect sizes for symptom improvement.

• Those studies with 50% maximum heart rate had lower attrition and better symptom improvement than those with the higher intensity.

• Higher intensity studies resulted in greater fitness gains compared to lower intensity in subjects who could complete the intervention.

• Subjects attained symptom relief, particularly decreased pain and fatigue as well as improved sleep and mod, with low to moderate intensity exercise of any type. Even very low movement therapies, such as QiGong, had significant symptom improvement.

• Strength and flexibility training are beneficial for symptom control and fitness improvements but there are insufficient data for recommending a uniform, evidence-based prescription for either of these modalities.

• Descriptive data as well as exercise intervention studies in men, minorities, children and older adults with FM are lacking. The fitness gains in older subjects were comparable to gains seen in age matched healthy controls and were significant compared to the subject's own baseline scores.

• No FM intervention to date has included only overweight or obese persons or individualized the intervention to their unique movement needs (e.g., lower extremity joint protection during weight bearing, awareness of comorbidities such as plantar fasciitis, ankle tendonitis, knee osteoarthritis and a myriad of psychological stigma regarding appearance).

• There is a lack of couples or family based exercise studies in FM, though these are common in healthy elderly, heart disease and other chronic illnesses [17-19].

### Recommendations for future research

• Determine optimal dosing of exercise so that an evidence based exercise prescription that includes mode, intensity, duration and frequency can be recommended.

• Determine the dose of exercise that effectively manages symptoms versus the dose that produces a symptom flare. This flare is more pronounced that the well documented delayed onset muscle soreness experienced by health deconditioned persons without FM who engage in unfamiliar muscle activity [20,21].

• Systematically tract the actual amount of exercise performed compared to the prescribed amount of exercise based on study protocol. Summarize and report these deviations in publication to help identify subgroups of FM patients that are unable to achieve a given level of activity.

• Select uniform symptom and outcome measures for FM exercise trials. Ideally symptoms could be monitored in "real time" rather than retrospectively. This approach would minimize recall bias and allow tracking of symptom trajectory over time. Calling subjects on some type of routine basis or having subjects carry a preprogrammed electronic device that alarms at set intervals requesting real time symptom data would be two ways to do this [22,23]. Outcome measures should include a patient graded global improvement score as is common in FM medication trials and recommended by the OMERACT 7 workshop [24].

• Examine the combined role of medications and exercise. Many FM subjects take medications and are told to exercise, yet only three studies thus far compare the combined and separate roles of exercise plus specific medications in FM [25-27], although many more acute dosing/cross sectional trials of drug and exercise in FM have been reported. At minimum, medications should be monitored and their use considered in statistical analyses.

• Include cost-utility analysis of exercise as a treatment for FM in future trials.

• Integrate families or other support systems into lifestyle interventions such as exercise as a way of improving long-term compliance.

• Test exercise modalities and movement therapies for a broader array of physical and mental health outcomes, beyond symptoms and physical fitness. For example, descriptive studies have found deficits in balance and increased falls in FM patients [28-30], yet only one intervention study measured balance as an outcome [54].

• Maximize methodological rigor. Randomization should be applied whenever possible to equally distribute variance throughout the groups. Hypotheses should be stated *a priori *and tested with appropriate correction for multiple comparisons and covaried for baseline differences between groups. CONSORT guidelines for reporting findings should be followed [31].

• Report compliance by calculating the number of classes or minutes attended divided by the number offered. Reporting compliance is critical as it allows reviewers to calculate the "dose" of the intervention that the subject actually received, similar to a pill count in a medication study.

• Conduct larger, longer-lasting RCTs that follow the individual from low impact exercise (e.g. pool settings to group based, land laboratory settings to home exercise with weekly booster sessions in community-based venues. This approach would better simulate a real-world application of exercise.

• Evaluate methods to increase compliance in longer trials to test techniques such as motivational interviewing.

## Competing interests

The author(s) declare that they have no competing interests.

## Authors' contributions

KDJ conceptualized this paper, analyzed the retrieved literature, and wrote the first draft. DA retrieved and analyzed literature and made the first draft of the table. KW retrieved literature, coauthored the first draft of the table and co-wrote the findings and recommendations sections. CSB critically reviewed and revised the original manuscript and co-wrote the findings and recommendations sections. All authors read and approved the final manuscript.

## Supplementary Material

Additional file 1 Dupree Jones. Table 1: Overview of 46 Exercise Intervention Studies for Subjects With Fibromyalgia (FM) (1988–2005) Click here for file
