# Supplementary material for: A comprehensive review of 46 exercise treatment studies in fibromyalgia (1988–2005)
Source: Health Qual Life Outcomes. 2006 Sep 25;4:67. doi: 10.1186/1477-7525-4-67 (PMC1590013; doi:10.1186/1477-7525-4-67)
Supplement: Additional file 1 — Dupree Jones. Table 1: Overview of 46 Exercise Intervention Studies for Subjects With Fibromyalgia (FM) (1988–2005) [file 1477-7525-4-67-S1.doc]

**Table 1: Overview of 46 Exercise Intervention Studies for Subjects With Fibromyalgia (FM) (1988—2005)**

| *Reference* | ***Design***  ***Subjects (n=enrolled, gender, age range)*** | ***Exercise Mode Program length in weeks (PL)*** | | | ***Duration (minutes per session)***  ***Frequency (times per week)***  ***Aerobic Intensity*** | | ***Attrition*** | | ***Outcome Measures*** |
| --- | --- | --- | --- | --- | --- | --- | --- | --- | --- |
| 1988 Study |  |  | | |  | |  | |  |
| 1) McCain et al. [32] | RCT  42 (gender undisclosed)  Age 27-54 | 2 groups:   1. Stationary bicycle 2. Flexibility   PL – 20 weeks | | | 60 minutes  3/week  THR 150 beats per pulse meter | | 14% Bicycle  5% Flex  11% Total | | 2-stage heart rate analysis  VAS pain, Total Myalgic Score, pain threshold, patient and physician global assessment |
| **Results/Conclusions/Comments**: The experimental group improved significantly in cardiovascular (CV) fitness, pain threshold levels and patient/physician global assessment. This was the first study to show that persons with FM who undergo aerobic training can increase CV fitness although FM symptoms of poor sleep and pain did not improve. Attrition was higher in the cycling group giving early indication that cycle ergometry might be poorly tolerated due to gluteal tender point activation. | | | | | | | | | |
| **1992 Studies** |  | |  |  | |  | |  | |
| 2) Høydalsmo et al. [33] Abstract only | Single Group  Age and gender not disclosed | | Multidisciplinary training program, to increase aerobic, fitness and flexibility  PL – 4 weeks | 45 minutes  5/day  (Daily total 3.5 hrs) | | Not reported | | V0_2max_ by cycle ergometry, tender points | |
| **Results/Conclusions/Comments:** This study demonstrated that aerobic and flexibility training improved aerobic capacity and decreased the number of tender points. Like the McCain study, fitness improvements were significantly more pronounced than symptom improvements, perhaps indicating that the frequency and duration of the exercise were too high. | | | | | | | | | |
| 3) Isomeri et al. [25] Abstract only | RCT  45 (39 females, 6 males)  Age 24-55 | | 3 groups:   1. Amitriptyline and light physical training, 2. Aerobic training and amitriptyline 3. Aerobic training only   PL – Hospital for 3 weeks then home for 12 weeks | Duration, frequency and intensity unknown | | Not reported | | VAS Pain, tender points | |
| **Results/Conclusions/Comments:** VAS measures of pain improved in all groups at 3 weeks. As this study was only reported in abstract form, whether the analyses were within group or between groups is unknown. Only the amitriptyline and aerobic training group had significant improvements at 3 months. Thus, a combination of drug and exercise was superior to either alone. The results were potentially confounded by variation in home and workloads after the initial period of hospitalization. | | | | | | | | | |
| 4) Mengshoel et al. [34] | RCT  35 females  Age 21-42 | | 2 groups:   1. Low impact aerobics 2. Treatment as usual controls (TAU)   PL – 20 weeks | 60 minutes  2/week  THR 120-150 beats/minute (pulse watch recorder) | | 39% Treatment  18% Control  29% Total | | Cycle ergometry heart rate at steady state, VAS pain | |
| **Results/Conclusions/Comments**: Aerobic dance improved aerobic fitness in the treatment group, but symptoms (pain, fatigue, coping) did not improve. The high attrition rate may have resulted in a sample size was too small to overcome baseline variability. The fact that fitness measures improved but symptoms did not and that the treatment group had the highest attrition may indicate that the intensity was too high. | | | | | | | | | |

| 1994 studies | |  |  |  |  |  |
| --- | --- | --- | --- | --- | --- | --- |
| 5) Burckhardt et al. [35] | RCT  99 females  Age 24-69 | | 3 groups:   1. Education plus physical training 2. Education 3. Delayed treatment control   PL – 6 weeks | Group 1:  60 minutes  1/week  Intensity unknown  Groups 1 and 2 received 90 minutes of education weekly | 15% Education + training  10% Education  14% Control  13% Total | FIQ, Quality of Life Scale, Self-efficacy scale, 6 minute walk, flexibility, chair test, Tender points |
| Results/Conclusions/Comments: Quality of life and self-efficacy were significantly better in groups 1 and 2 when compared to group 3 at posttest. Other changes were smaller and more delayed than had been expected. The study concluded that a longer education program, more vigorous physical training, and long-term follow-up were needed. This study was limited by education about how to exercise rather than direct supervision of exercise. The lower attrition rate compared to previous studies was the first indication that allowing subjects to determine their own level of intensity may be preferable in the long run. This study was the first to measure the outcome variables on follow-up (24 weeks). | | | | | | |
| 6) Nichols et a1. [36] | RCT  24 (22 females, 2 males)  Age 30-69 | | 2 groups:   1. Treatment - stretching, 2 pool classes, individualized aerobic activity (walking, swimming or bicycling) 2. Sedentary controls   PL – 8 weeks | 20 minutes  3/week  THR 60-70% (based on age and gender norms)  Predicted HR by second week calculated by palpitation | 20% Treatment  33% Control  21% Total | McGill Pain Questionnaire,  Sickness Impact Profile (SIP), Brief Symptom Inventory (BSI) |
| **Results/Conclusions/Comments:** The experimental group demonstrated significantly better scores on the positive symptom total of the BSI but significantly worse scores on the physical dimension of the SIP compared to the controls at posttest. There were trends in the pain ratings suggestive of a beneficial response to aerobic walking. This lack of benefit on symptoms was most likely due to the short duration of the study, 8 weeks, intensity and frequency of the aerobic activity. The study provided more evidence that FM exercise studies need to be longer than those in the general population for benefits to be seen if the intensity is lower. | | | | | | |
| 1995 studies |  | |  |  |  |  |
| 7) Clark  et al. [37] Abstract only | RCT  30 females  Age not  disclosed | | 2 groups:   1. Aerobic cycling 2. Sedentary control   PL- 12 weeks | 30 minutes  3/week  75% predicted heart rate | 20% Exercise  27% Control  23% Total | V0_2max_, Total Myalgic Score, global assessment, quality of life |
| **Results/Conclusions/Comments**: Subjects were able to exercise at a training index sufficient to produce changes in aerobic capacity (average 20% change). Those who could tolerate the intervention improved pain and global assessment scores. This is the first study that resembled a dose/response trial (intensity x duration x frequency). Attrition was higher than the McCain study, possibly indicating that cycle ergometry was painful for many subjects. | | | | | | |
| 8) Hannonen et al. [38] Abstract only | RCT  40 females  Age not disclosed | | 2 groups:   1. Aerobic training 2. Muscle strengthening   PL - 24 weeks | Duration, frequency and intensity not disclosed | 0% Aerobic group  10% Muscle strengthening | V0_2max_, trunk flexors and extensor strength, Modified Health Assessment Questionnaire, Nottingham Health Profile |
| **Results/Conclusions/Comments:** Aerobic training produced statistically significant within group changes in V0_2max_ at 6 months and one year. Muscle strengthening produced statistically significant within group changes in strength at 6 months and one year. Both groups experienced statistically significant improvements in pain. These data were the first to suggest that people with FM could add strength training to their exercise protocol although the protocol was limited by self-report rather than direct supervision. | | | | | | |
| 9) Mengshoel et al. [39] Abstract only | Single group  16 women  Age not disclosed | | Multidisciplinary exercise and cognitive behavioral therapy (CBT)  PL - 10 weeks | Duration, frequency and intensity not disclosed | Not disclosed | General pain intensity |
| **Results/Conclusions/Comments:** Pain was reduced significantly at the end of the intervention and maintained at 6 months post-intervention. This is a small study limited by the single-group design. | | | | | | |

| 1996 studies |  |  |  |  |  |
| --- | --- | --- | --- | --- | --- |
| 10) Martin et al. [40] | RCT  60  Age 34-56 | 2 groups:   1. Exercise (aerobic walking, strength training (universal   gym and surgical tubing), flexibility   1. Relaxation and yoga   PL – 6 weeks | 60 minutes  3/week  THR 60-80%  For weight training  # sets and repetitions not disclosed | 40% Exercise  33% Relaxation  37% Total | V0_2max_, isokinetic dynamometer (flexion and extension knee) internal and external rotation of the shoulders, sit and reach box, Total Myalgic Score, Tender Points, VAS pain, FIQ |
| **Results/Conclusions/Comments**: Exercise training produced significant improvements over relaxation for sit-and-reach scores, myalgic score, number of tender points, and V0_2max_ at 6 weeks. Symptom and fitness improved in this short study. Attrition was high in the exercise group probably because of high aerobic intensity. No explanation is offered for the high attrition on the relaxation/yoga group and no description of the yoga poses was given. | | | | | |
| 11) Wigers et al. [41] | RCT  60 (55 females, 5 males)  Age 23-73 | 3 groups:  1. Aerobic exercise  2. Stress management  3. Treatment as usual controls  PL – 14 weeks, exercise  PL – 8 weeks stress management | Exercise:  45 minutes  3/week  THR 60-70% AGN  Stress management:  90 minutes  1-2/ week | 25% Aerobic  33% Stress management  18% Treatment as usual controls  25% Total | Work capacity bicycle ergometry, dolorimetry, VAS for pain, fatigue, disturbed sleep, depression, Global subjective improvement |
| **Results/Conclusions/Comments**: Both the stress management and aerobic exercise group had significant improvements on pain and dolorimetry scores when compared to the control group at treatment completion. The aerobic exercise group was also significantly improved on pain distribution, energy and work capacity. The stress management group was significantly improved on depression when compared to controls. At the 4.5-year follow-up of the treated groups, the aerobic exercise subjects who continued exercising were significantly better on pain, energy, depression and work capacity than those who had discontinued exercise. This is the first study with a stated effort to minimize eccentric muscle work, which is known in other populations to worsen delayed onset muscle soreness. This study demonstrated that long-term compliance to exercise without supervised sessions is difficult to maintain. | | | | | |
| 1997 study |  |  |  |  |  |
| 12) Nørregaard et al. [42] | RCT  38 (gender not disclosed)  Age 32-65 | 3 groups:   1. Aerobic dance (running and fast paced movements at 40-50% V0_2max_ ) 2. Steady exercise (free standing gymnastic exercise performed a steady speed) 3. Hot packs   PL – 12 weeks | Aerobic dance:  50 minutes  3/week  Steady exercise:  50 minutes  2/week  Hot packs:  30 minutes  2/week  HR 40-50% AGN | 67% aerobic dance  27% steady exercise  13% hot pack  39% total | Bicycle ergometry for work capacity, dynamometer for quads and biceps, VAS pain and fatigue, FIQ, tender points, Beck Depression Inventory (BDI) |
| **Results/Conclusions/Comments:** There were no significant differences between or within the groups on any measured outcome. The aerobic group, suffered from the worst attrition of any of the 3 groups, perhaps because running and high impact movements comprised a major portion of the treatment protocol. This study suggests that the mode of exercise should fit the physical capabilities of the subjects. The physical limitations of the average FM patient were well known in the literature by this time but seem to have been unappreciated by the researchers in this study. There may have been an interaction effect between the method of recruitment and the intensity of the exercise. | | | | | |

| 1999 studies |  |  |  |  | |  |
| --- | --- | --- | --- | --- | --- | --- |
| 13) Gowans et al. [43] | RCT  41 (36 females, 9 males)  Age 24-72 | 2 groups:   1. Water aerobic, strength and flexibility exercise plus education 2. Wait listed control group   PL – 6 weeks | Exercise:  30 minutes  2/week  Education:  120 minutes total  THR 60-70% AGN | 13% Treatment  5% Control  9% Total | | 6 minute walk, FIQ Arthritis Self-efficacy Scale |
| **Results/Conclusions/Comments:** Water aerobics plus education produced significant improvements in the 6-minute walk, well-being, and fatigue at 3 months when compared to controls. This is the first water-based FM exercise intervention. The benefits of the combined program may have been due to exercise or education since both interventions were given simultaneously. | | | | | | |
| 14) White et al. [44]  Abstract only | Single group  8 (6 females, 2 males)  Age mean 49 years | Warm water aerobics, flexibility and strength.  PL – 6 weeks | 60 minutes  3/week  THR 70% | 25% Total | Treadmill V0_2max_, 6-minute walk, FIQ, Tender points | |
| **Results/Conclusions/Comments**: The subjects improved on the 6-minute walk and tender points but not the total FIQ score or V0_2max_. This was the 2^nd^ water-based study in FM and further demonstrated the feasibility of this type of exercise. | | | | | | |
| 2000 studies |  |  |  |  |  | |
| 15) Creamer et al. [45] | Single group  28 (27 females, 1 male)  Age 37-59 | Education, behavioral therapy, relaxation, QiGong  PL – 8 weeks | 150 minutes  1/week  Intensity not disclosed | 29% Treatment | FIQ, SF-36, Beck Depression Inventory, Patient Activity Recall, Tender points, Heath Assessment Questionnaire, 6-minute walk test, RAND Health Survey | |
| **Results/Conclusions/Comments:**  Significant improvements were seen on the FIQ, HAQ, BDI, patient global, 6-minute walk, number of tender points, mean pain threshold, and the RAND Health Survey. All improvements with the exception of the BDI were sustained at 4 months post-intervention. This is the first FM study focus on mind-body interactions and use QiGong movements. Validity was limited by a lack of control group. | | | | | | |
| 16) Mannerkorpi et al. [46] | RCT  58 (gender not disclosed)  Age 35-59 | 2 groups:   1. Warm water exercises plus education 2. Education control   PL – 24 weeks | Pool:  35 minutes  1/week  Intensity not disclosed  Education:  60 minutes  1/week for 6 weeks | 24% Treatment  6% Education control | FIQ, 6 minute walk, test for functional limitations, symptom severity, Quality of Life Scale, Arthritis Self-efficacy Scale | |
| **Results/Conclusions/Comments**: A 6-month program of exercise in a temperate pool combined with education resulted in significant improvements in total FIQ, 6-minute walk, grip strength, anxiety, physical function, pain severity, affective distress, and quality of life when compared with the control group at posttest. This study describes the water treatment in sufficient detail to be replicated. This is a very high quality study, high quality well written study. | | | | | | |
| 17) Meiworm et al. [47] | Non-randomized, controlled trial  39 (36 females, 3 males)  Age 35-57 | 2 groups:   1. Jogging, walking, cycling or swimming, at 50% V0_2max_ increasing at 2 weeks 2. Sedentary controls   PL – 12 weeks | 25 minutes  2-3/week  THR 115 beats/minute (corresponding to groups mean 50% V0_2max_) | 7% Exercise  0% Control | Spirometrics, dolorimetry, body pain diagram, VAS pain | |
| **Results/Conclusions/Comments:** Aerobic endurance exercise led to within-group improvements in aerobic fitness and pain. What is new about this study compared to previous ones is that the subjects chose their aerobic training method (jogging, walking, cycling, swimming or mixed). The experimental treatments are detailed enough to replicate. This may have led to the impressively low attrition rate of 7% in a 12-week study. There is no explanation for why the authors did not compare the experimental group to the control group. | | | | | | |

| 18) Meyer and Lemley [48] | RCT  21 females  Age 43-56 | 3 groups of home- based individual  walking exercise Groups:   1. High intensity (40% MRH progressing to maximum 85% in week 5) 2. Low intensity (25% MHR progressing to 60% after week 10) 3. Control group   PL – 24 weeks | 10-30 minutes  3/week  THR 50% through week 9  60% max after week 10  High intensity increase 10% per week until week 4 and then up to 85% after week 5  HR monitoring technique not disclosed | 62% Total | Tender points, heart rate, perceived exertion, FIQ, Beck Depression Inventory, VAS pain, disabil­ity index |
| --- | --- | --- | --- | --- | --- |
| **Results/Conclusions/Comments**: There were no significant differences in the groups at the end of the study. Only 8 subjects completed the 24 weeks. The authors reanalyzed their data with the 8 subjects by dividing them into two groups based on actual heart rate intensity attained during the study. This analysis found that pain influenced the intensity at which the subjects were willing to exercise. Pain score were worse in the high intensity completer group perhaps indicating that people with FM may experience a pain flare when working at intensities that are tolerated by the general population. | | | | | |
| 19) Ramsay et al. [49] | RCT  74 females  Age not disclosed | 2 groups:   1. One time demonstration and written instructions for home aerobic, strength and stretching, with unknown number of unsupervised home visits 2. Once weekly supervised aerobic, strength and stretching with instructions to exercise at home   PL – 12 weeks | 60 minutes  1/week supervised  Intensity not disclosed | 5% Group 1  59% Group 2  32% Total | Resting pulse, VAS pain, Total Myalgic Score, number of nights/week poor sleep, average hours sleep/night, Health Assessment Questionnaire |
| **Results/Conclusions/Comments:** The only significant finding was an improvement in anxiety in the supervised exercise group compared to the unsupervised group. At 24 and 48 weeks follow-up, trends toward improvement were not sustained. The continuation rate of exercises at home was low in both groups. This study is important because individualized physical therapy instructions to exercise at home is the standard of care in the U.S. for exercise in FM, as evidenced by reimbursement by 3^rd^ party payers for physical therapy but not group exercise classes. | | | | | |
| 2001 studies |  |  |  |  |  |
| 20) Gowans et al. [50] | RCT  57 gender not disclosed  Age 36-57 | 2 groups:   1. Treatment at three levels: a) water and then aerobics gradually increased to 60-75% maximum heart rate b) then 30 minutes 2 times week of just aerobics for 17 weeks c) then 30 minutes aerobics once a week for 17 weeks 2. Treatment as usual control   PL – 23 weeks | Treatment levels  a) 30 minutes  3/week  b) 30 minutes  2/week  c) 30 minutes  1/week  THR 60-70% AGN | 41% Exercise  0% Control  21% Total | 6 minute walk, FIQ, Beck Depression Inventory, Tender points, isometric knee strength, State-Trait Anxiety Inventory (STAI), Arthritis self efficacy scale (ASES) |
| **Results/Conclusions/Comments:** There were significant improvements in 6-minute walk distance, BDI, FIQ, ASES, STAI and MHI in the experimental group subjects compared to the control group subjects who met efficacy criteria. This is the first study that began with water exercise and progressed to land-based exercise. | | | | | |

| 21) Häkkinen et al. [51] | RCT  21 females with FM  12 females as healthy controls  Age 31-45 | 3 groups:   1. Muscle strengthening with machine weights 2. FM treatment as usual controls   3. Healthy training control  PL – 21 weeks | Duration unknown 2/week  Intensity of weight training  15-20 reps of 6-8 sets @ 40-60% 1-RM for 3 weeks progressing to 5-10 reps, 6-8 sets @ 70-80% 1-RM | 0% Total | % Body fat, tender points, strength (concentric, isometric and vertical squat jump), VAS pain and sleep, Beck Depression Inventory, Health Assessment Questionnaire surface (EMG) activity, maximal unilateral isometric force of the right knee extensors, and muscle cross-sectional area of the quadriceps femoris, serum total and free testosterone, growth hormone (GH) insulin-like growth factor |
| --- | --- | --- | --- | --- | --- |
| **Results/Conclusions/Comments**: Strength training adaptation measured by maximal dynamic and isometric muscle strength and iEMG was comparable in matched FM and healthy controls. Pain was not significantly improved although fatigue and depression were improved in the FM treatment group compared to the FM control group. Progressive strength training can safely be used in the treatment of FM to decrease the impact of the syndrome on the neuromuscular system, perceived symptoms, and functional capacity. These results support the opinion that FM has a central rather than a peripheral or muscular basis. Improvements in perceived symptoms and function support the assumption that even heavy resistance strength training can safely be used in the treatment of patients with FM. | | | | | |
| 22) Jentoft et al. [52] | RCT  44 females  Age 31-52 | 2 groups:   1. Aerobics, strengthening and stretching on land 2. Pool   PL – 20 weeks | 60 minutes  2/week  THR 60-80% AGN by pulse watch recorder | 27% Land  18% Pool  23% Total | FIQ, Arthritis Self Efficacy Scale, grip strength, fatigue, V0_2max_ |
| **Results/Conclusions/Comments:** The land group had significant improvements in grip strength when compared to the pool group. No between groups differences on symptoms or self-efficacy were found. Significant improvements in cardiovascular capacity, walk time, fatigue and stiffness were found in both groups. The pool group demonstrated greater improvement in pain, anxiety and depression. Physical capacity can be increased by exercise, even in warm-water. This small, but well designed study demonstrated the efficacy of both types of exercise. It also suggested that pool exercise might be better at symptom relief. | | | | | |
| 23) Martin et al. [53]  Abstract only | RCT  111 (107 females, 4 males)  Age not disclosed | 2 groups:   1. Aerobic, flexibility and muscle strengthening 2. Same exercise and self-management education   PL – 6 weeks | 60 minutes  3/week  Group 2 an additional 1 hour seminar  Intensity not disclosed, muscle strength, mode and number of reps not disclosed | 7% Total | 6-minute walk, dynamometer, sit and reach flexibility box, FIQ, Tender points, Total myalgic score, illness intrusiveness, self-efficacy scale |
| **Results/Conclusions/Comments**: The authors report significant differences by group on strength measures, 6-minute walk and flexibility. However, there is insufficient data to determine if the addition of self-management is effective. | | | | | |
| 2002 studies |  |  |  |  |  |
| 24) Jones et al. [54] | RCT  68 females  Age 38-56 | 2 groups:   1. Supervised group stretching with surgical tubing 2. Supervised group muscle strengthening with surgical tubing and hand weights   PL – 12 weeks | 60 minutes  2/week  Strength 3 reps x 12 sets increased at subject’s discretion | 7% Total | Shoulder and quadriceps strength by isokinetic dynamometry, internal and external shoulder rotation for flexibility, Tender points, Total Myalgic Score, VAS pain, FIQ, Beck Depression Inventory, |
| **Results/Conclusions/Comments:** Muscle strengthening showed significant improvements and greater magnitude of change in 14 measures compared to 7 measures improved in stretching. It is feasible for FM subjects to take part in a specially tailored muscle-strengthening program that minimized eccentric muscle work and provided pauses in appreciation for delayed return to baseline state. Subjects experienced an improvement in overall disease activity, without a significant exercise induced flare in pain. Flexibility training alone also results in overall improvements, albeit of a lesser degree. Improvements were sustained at 1 year in the strength group only. This study isolated muscle strengthening, as did Hannonen in 1995. It was the first to demonstrate symptom improvement with strengthening alone. Attrition was low perhaps due to both the specificity of the exercises and self-efficacy enhancement through group work. | | | | | |

| 25) King et al. [55] | RCT  196 females  Age not disclosed | 4 groups:   1. Aerobics 2. Education 3. Aerobics and Education 4. Treatment as usual controls   PL – 12 weeks | Aerobics:  20-40 minutes  3/week  60% AGN by pulse  Education:  1.5-2 hours per session  1/week  Aerobics + Education:  Aerobics same as group 1, but then on 3^rd^ day had the educational component | 22% Total | 6-minute walk, FIQ, Quality of Life, Chronic Self-Efficacy Scale |
| --- | --- | --- | --- | --- | --- |
| **Results/Conclusions/Comments:** This study demonstrated that subjects who complied with the treatment protocol improved their perceived ability to cope with other symptoms and aerobic fitness. The authors rigorously disclosed the limitations of the study. They also adroitly describe the difficulties in completing RCTs in an FM population and made important recommendations for future studies. This paper models the CONSORT reporting guidelines. | | | | | |
| 26) Richards and Scott [56] | RCT  136 (126 females and 10 males)  Age 18-70 | 2 groups:   1. Individual aerobic exercise, walking, cycling on exercise bikes 2. Relaxation   PL – 12 weeks | 60 minutes  2/week  Intensity by talk test | 21% Total | Global self-rating of improvement, Tender points, FIQ, McGill short form, SF- 36 |
| **Results/Conclusions/Comments:** Between group and within group scores were not significant at 3 months on any variable except global self-rating score. At 6 months post intervention, symptom improvements were statistically significant in aerobic compared to relaxation training. All statistical significance was lost by 12 months post intervention, indicating a need to develop programs that subjects can adopt as part of long-term lifestyle change. | | | | | |
| 27) Rooks et al. [57] | Single group  24 females  Age 36-54 | Pool 4 weeks, then cardiovascular aerobics, strength training and flexibility  PL – 12 weeks | 60 minutes  3/week  Subjects measured their pulse rates but THR not disclosed | 38% total | FIQ, muscle strength by 1-RM, 6-minute walk |
| **Results/Conclusions/Comments:** Total FIQ, 1-RM leg press, chest press, FIQ, VAS for stiffness and anxiety were significantly improved. This feasibility study demonstrated that pool-based exercise progressing to land-based exercise can be a safe, well tolerated, and effective strategy for improving muscle strength, cardiovascular endurance and functional status in women with FMS without exacerbating symptoms. | | | | | |
| 28) van Santen et al. [58] | RCT  143 females  Age 26-60 | 3 groups:   1. Fitness (high impact, supervised, small group intense aerobic exercise, strengthening and stretching) 2. Biofeedback (individually applied, supervised, contraction relaxation with electronic biofeedback apparatus) 3. Treatment as usual controls   PL – 24 weeks fitness  8 weeks biofeedback | Fitness training:  60 minutes  2/week (half of the fitness training group were offered once weekly 90 min FM education classes  No one exceeded 130 beats/minute  Biofeedback:  30 minutes  2/week | 18% Total | Tender points, Total Myalgic Score, bicycle ergometry, Borgmax, fatigue, distress, Arthritis impact measure scale, physical fitness, Sickness Impact Profile, patient global assessment |
| Results/Conclusions/Comments: Intent to treat analyses found no differences between the 3 groups at follow up on symptoms or fitness measures. Further analysis of the subgroup of subjects with a high attendance rate (> 67%) also showed no improvement. Lack of significance may have been due in part to high variability in the outcome markers. Worsening scores on cycle ergometry may have been related to subject’s desire to avoid post exercise pain. This study highlights the problem of heterogeneity in both the FM population and commonly employed outcome markers. It also underscores FM patient’s aversion to pain inducing high intensity exercise either during testing or treatment. | | | | | |

| 29) van Santen et al. [59] | RCT  37 females  Age 20-56 | 2 groups:   1. High-intensity fitness training consecutive weeks (strength, stretch, and stationary cycling) 2. Low-intensity fitness training (strength, stretch, and aerobic dance)   PL – 20 weeks | High intensity:  60 minutes  2-3/week  Maintenance of 150 beats/minute for 20-30 minutes  Low intensity:  60 minutes  3/week  Intensity not disclosed | 11% High impact  28% Low impact  19% Total | Patient global assessment, VAS Pain, Tender points, Wmax (physical fitness), health status, Total Myalgic Score, health status, physical fitness, psychological distress |
| --- | --- | --- | --- | --- | --- |
| **Results/Conclusions/Comments**: There was no difference between the two groups on any variable. Dropouts and non-compliers cited pain and fatigue induced by exercise. The high intensity group reported feeling “completely broken down” (p 586) after each exercise session. There was now mounting evidence that higher intensity, higher impact exercise worsens symptom scores. Low intensity exercise may be more effective in the long-term | | | | | |
| 2003 studies |  |  |  |  |  |
| 30) Astin et al. [60] | RCT  128 (127 females, 1 male)  Age 37-60 | 2 groups:   1. Mindfulness meditation plus QiGong movement 2. Education control   PL – 8 weeks | 150 minutes  1/week  Education Control:  150 minutes  1/week | 50% Treatment  48% control  49% Total | Tender points, Total Myalgic Score, FIQ, 6-minute walk, Beck Depression Inventory |
| Results/Conclusions/Comments: Both groups improved on FIQ, total myalgic score, pain and depression but not on the 6-minute walk. There was no evidence that the multimodal mind-body intervention for FM was superior to education. Attrition was very high for a non-aerobic study. It is possible that the 2.5-hour class duration was too long for FM subjects. This study underscores the need to maximize differences between the treatment and control group or consider non-education, non-activity based controls for future exercise studies. | | | | | |
| 31) de Assis et al. [61] Abstract only | RCT  60 females  Age 18-60 | 2 groups   1. Deep water running (harness suspension, supervised, 29^o^C) 2. Land-based exercise (walking or jogging)   PL – 15 weeks | Duration, frequency and intensity not disclosed | 13% Treatment  13% total | VAS, FIQ, Beck Depression Inventory, SF-36, global improvement scale |
| **Results/Conclusions/Comments**: There were no between group differences on VAS, Global improvement, BDI, SF-36 total and aerobic fitness by oxygen uptake peak. Within group differences were significant for pain improvement in both groups. Deep water running in a warm swimming pool was more effective than land-based exercise in emotional aspects of the SF-36. This study stated that handicapped persons might potentially benefit from deep water running. This study, published only as an abstract to date, may have hit upon the emotional aspects of helping people be successful in their attempts to exercise, which has implications for bolstering self-efficacy with realistic exercise prescriptions. | | | | | |
| 32) Schachter et al. [62] | RCT  143 females  Age 20-55 | 3 groups:   1. Long bout 2. Short bout 3. No exercise   PL – 16 weeks | **Long bout**  10-/week  **Short bout**  5-15 minutes – twice a day  3-5/week  Intensity 40%-50% increasing to 65-75% of THR, via self measured heart rate and perceived exertion. | 29% Long bout  38% Short bout  14% No exercise  29% Total | Physical function, FIQ, VAS pain, Tender points, Self efficacy |
| **Results/Conclusions/Comments:** Progressive, home-based, low-impact aerobics improved physical function and FM symptoms minimally in participants who completed at least two thirds of the recommend exercise. Fractionation of exercise training provided no advantage in terms of exercise adherence, improvements in FM symptoms of physical function. High attrition rates and problems with exercise adherence were experience in both exercise groups. Follow-up attrition problems were greatest in the treatment groups. The authors concluded that a 16-week home-based videotaped program of aerobics had beneficial effects on disease severity and the long bout format improved fitness. This study tested the benefits proposed by the U.S. Surgeon General’s guidelines for cumulative short bout exercise for sedentary persons. | | | | | |

| 33) Taggart et al. [63] | Single group  37 (35 females, 2 males)  Age 26-80 | 1 group T’ai Chi  PL – 6 weeks | 60 minutes  2/week  Duration of stretches and types not disclosed | 43% Total | FIQ, SF-36, Health Assessment Questionnaire |
| --- | --- | --- | --- | --- | --- |
| **Results/Conclusions/Comments:** Statistically significant improvement in the FIQ and SF-36 were found. T’ai Chi was potentially beneficial to subjects with FM. Study was limited by absence of a control group. However, it provided effect sizes for commonly used FM outcomes so that a randomized controlled clinical trial in T’ai Chi is warranted. It is also possible that the T’ai Chi exercises should be tailored to decrease length of time set in poses, minimize overhead work and allow for alternative balance poses; tailoring the exercises may have improved attrition/compliance. | | | | | |
| 34) Valim et al. [64] | RCT  76 females  Age 18-65 | 2 groups:   1. Aerobics 2. Flexibility   PL – 20 weeks | Duration, frequency and intensity not disclosed | 16% Aerobic  26 % Flexibility  21% Total | FIQ, SF-36, VAS pain, Beck Depression Inventory |
| **Results/Conclusions/Comments:** Aerobic training was significantly superior to flexibility training for increasing aerobic fitness and decreased FM symptoms. This study was very similar to the first exercise intervention in FM (McCain). This exercise study is unique in that it controlled for medication use, allowing only Tylenol. Lack of medication for symptom control may have contributed to attrition. Also, this study confirmed that fitness gains do not correlate with symptom changes. | | | | | |
| 2004 studies |  |  |  |  |  |
| 35) Altan et al. [65] | RCT  50 females  Age 31-56 | 2 groups:   1. Water exercise (walking, jumping active ROM, stretching and relaxation and slow swimming) in warm mineral water pool 2. Warm water pool balneotherapy only   PL – 12 weeks | 35 minutes  3/week  Intensity not disclosed | 4% Group 1  12% Group 2  8% Total | VAS, FIQ, Total Myalgic Score, Tender Points, Beck Depression Inventory |
| Results/Conclusions/Comments: The results showed that both treatments significantly improved pain and other FM symptoms at 12 and 24 weeks. There were no significant differences between the groups on any variable with the exception of the BDI. This study suggests that balneotherapy is a valid treatment option in FMS. This study was administered in Turkey where balneotherapy is common. Balneotherapy may help break the pain-immobility problem experienced by many persons with FM and may be used as a pre-conditioning treatment prior to more active exercise treatments in future studiess. | | | | | |
| 36) Cedraschi et al. [66] | RCT  164 (152 females, 12 males)  Age 39-59 | 2 groups:   1. Swimming, relaxation exercises land-based exercises, daily activity sessions, discussion sessions 2. Wait-listed controls   PL – 6 weeks | 90 minutes  2/week  Intensity not disclosed | Treatment 21%  Control 19%  Total 21% | Tender Points, Total Myaglic Score, Quality of Life Scale, patient satisfaction, pain score diagrams, FIQ, Psychological General Well-Being Index, patient satisfaction measures |
| **Results/Conclusions/Comments:** The exercise group had improved quality of life and function compared to control. Improvements were maintained for 6 months after completion of program. Attrition and full participation were problematic for such a short study, but it was adequately powered to overcome drop out. Results are to be interpreted with caution as multiple t-tests were used for analyses without disclosure of correction for multiple comparisons. | | | | | |
| 37) Fontaine 2004 et al. [67] Abstract only | RCT  29 (gender not disclosed)  Age not disclosed | 2 groups:   1. Lifetime physical activity exercises (LPA) 2. FM education   PL – 12 weeks | 30 minutes of accumulated activity  5-7/week  Intensity not disclosed | 28% Total | 6-minute walk, FIQ, fatigue, severity scale |
| **Results/Conclusions/Comments:** No between group differences were reported except improvements in self-perception of health status in the exercise group. The 12-week LPA intervention produced a 59% increase in physical activity compared to baseline based on activity logs and pedometers. Accumulating 30 minutes of moderate intensity LPA in short bouts throughout the day significantly improved self-reported health status and had moderate, though non-significant effects on pain, fatigue, disability and performance. This lack of difference in FIQ and fatigue may have been due to application of symptom management techniques, social support and attention in the education group. These results build on those reported by Schachter comparing long and short bout exercise. | | | | | |

| 38) Redondo et al. [68] | RCT  31 females  Age not disclosed | 2 groups:   1. Cognitive behavioral therapy/education (CBT) 2. Mixed land and pool based aerobic, strength and stretching exercises   PL – 8 weeks | Exercise:  45 minutes  5/week  Intensity not disclosed  CBT:  150 minutes  1/week | 24% CBT  21% Exercise  23% Total | FIQ, Beck Depression Inventory, chronic pain self-efficacy scale and chronic pain coping inventory, physical activity, aerobic capacity, chronic Pain Self-Efficacy Scale, Chronic Pain Coping Inventory |
| --- | --- | --- | --- | --- | --- |
| **Results/Conclusions/Comments:**  The only between group difference was increased use of the relaxation strategy in the CBT group, but this was not an *a priori* hypothesized primary outcome variable. Within group changes included some FIQ items and coping strategies which improved in both groups; all measures of functional capacity improved in the exercise group while only one physical measure (vertebral column, strength, flexibility and pain) improved in the cognitive behavior group. Surprisingly no differences in anxiety, depression, and self-efficacy were found in either group. After one year, parameters returned to baseline in both groups, except functional capacity in exercise group remained better after one year. PE. Lack of between group differences may have also been produced by the short time of intervention (8 weeks, small sample size (15 and 16 completed per group) and multiple outcome measures. | | | | | |
| 2005 Studies |  |  |  |  |  |
| 39) Beltran and Gevirtz [69] Abstract only | RCT  34 females  Age 22-65 | 3 groups:   1. Multi-component treatment group, which included pool and psychoeducation 2. Exercise only, pool   Treatment as usual controls  PL – 10 weeks | 45 minutes exercise, 45 minutes psychoeducation  2/week  60-80% AGN by heart beat monitors | Multi-component 8%  Exercise 9%  Control 0%  Total 6% | Functioning, pain, sleep, fitness |
| **Results/Conclusions/Comments:** Significant positive changes in sleep were found in the multi-component group when compared with the other two groups. The authors collapsed the two treatment groups and then found significant improvements on functioning, fatigue, pain, sleep and stiffness. This finding should be interpreted with caution as the sample size is very small and the collapsed group is potentially confounded by the two subgroups receiving a different dose of treatment. | | | | | |
| 40) Da Costa et al. [70] | RCT  80 females  Age 41-56 | 2 groups:   1. Individualized home based moderate intensity exercise program 2. Usual care group + weekly attention by completing questionnaires   PL – 12 weeks | Individual prescription:  60-120 minutes per week. Duration dependent on the intensity of the activity.  Frequency individualized  THR 60-70% gradually increasing to 75-85% THR Used heart rate monitor | 28% Exercise  20% Control  24% Total | FIQ, BMI, VAS, baseline fitness maximum metabolic equivalents (METs), pain severity, psychological distress, Global Severity Index |
| Results/Conclusions/Comments: Results of this repeated measures study indicated that the exercise group had significant decreases in pain and FM impact over 9 months. The study did not use a repeated measures analysis, which might have provided stronger evidence for reported results since they would have been based on data for all time points combined. Home-based exercise, a relatively low-cost treatment modality, has the potential to improve important health outcomes in FM compared to usual care plus attention control. | | | | | |
| 41) Jones et al. [26] Abstract only | RCT  164 with FM (159 females, 5 males)  Age | 4 groups  1. Pyridostigmine and exercise  2. Pyridostigmine and diet recall   1. Exercise/diet recall 2. Placebo/diet recall   PL – 24 weeks | 50 minutes  3/week  Intensity by talk test | 5% Group 1  14% Group 2  0% Group 3  5% Group 4  6% Total | FIQ, VAS pain, growth hormone, V0_2max_, Total Myalgic Score, fitness with six standard tests including balance, GH, IGF-1 |
| **Results/Conclusions/Comments:** These results confirm that FM patients have a significant impairment of GH response to acute exercise, and that this is normalized by pyridostigmine. However, neither pyridostigmine alone nor pyridostigmine plus exercise normalized IGF-1 levels or improved most FM symptoms, with the exception of restorative sleep, fatigue and anxiety. The exercise groups significantly improved fitness including balance. We surmise that his lack of change in IGF-1 may be due to FM patient’s inability to exercise chronically at a high enough intensity to stimulate the GH response, and that improving IGF-1 levels in FM will probably require both an inhibition of somatostatin tone and stimulation of growth hormone releasing hormone. | | | | | |

| 42) Kingsley, et al. [71] | RCT  29 females  Age 18-54 | | 2 groups  1. Strength training  2. Wait list control  PL – 12 weeks | 30 minutes  2/week  Subjects performed 1 set of 8-12 repetitions. After 12 repetitions were performed with proper form, weight was increased by 2.3 to 4.5 kg (5-10 lb). Strength training for upper and lower body began at an intensity of approximate 30-40 repetitions of the subjects’ initial 1-RMs and progressed to 60-80% of the initial 1-RMs by week 12 | 7% Treatment  0% Control  3% Total | FIQ, strength, functionality, Tender Points, continuous scale physical for performance |
| --- | --- | --- | --- | --- | --- | --- |
| **Results/Conclusions/Comments:** Strength training improved strength and some functionality in women with FM compared to wait list controls. This study while important is potentially limited by small sample size and high attrition. Nonetheless, significant differences between groups were found with intent to treat analyses. The authors do not believe that attrition was related to exercise induced symptom flares, but we wonder if the 47% attrition at 4 weeks was related to the rapid increase in load. The authors instead state that psychological measures such as depression and anxiety may need to be considered as predictors of attrition. Pain medications were stable throughout the intervention, but no mention of other FM medication access or adjustment was mentioned (e.g., sleep or mood medication). This study also introduces a new scale to the FM exercise literature, the continuous scale physical for performance. This is an important tool to consider in future trials as deconditioning may lead to disability and dependence. This study, like previous others, demonstrated fitness improvements without symptom improvements. | | | | | | |
| 43) Lemstra and Olszynski [72] | | RCT  79  Age and gender not disclosed | 2 groups:   1. 18 supervised group submaximal aerobic exercise, stretch and weight training, 2 stress control/education lectures, 1 group dietary lecture, 2 massage sessions 2. Treatment as usual controls   PL - 6 weeks | Exercise:  10 – 30 minutes  3/week  Initially 50% maximum oxygen intake  Light weight training:  Beginning at 2 sets of 15 repetitions and 10#s increasing to the same repetitions ands sets but 30#s  3/week  Lectures:  180 minutes  1/week | 16% Treatment  0% Control  10% Total | Self-perceived health status, pain-related disability, average pain intensity, depressed mood, days in pain, hours in pain |
| Results/Conclusions/Comments: Between group changes were found in self-perceived health status, pain intensity, depression and days and hours in pain. No differences between groups were found in number and type of medication use. Positive health-related outcomes were obtained with low-cost, community-based, nonclinical setting. | | | | | | |

| 44) Salek et al. [27] | | RCT  68  Age and gender not disclose | 2 groups:   1. Aerobic exercise and tricyclic antidepressant 2. Tricyclic antidepressant control   PL - 16 weeks | Duration, frequency and intensity not disclose | Not disclosed | Pain grade, Tender points, occurrence or arousal at nigh, frequency of nocturnal micturition |
| --- | --- | --- | --- | --- | --- | --- |
| Results/Conclusions/Comments: There were no significant differences between groups on pre- versus post- measures. The exercise/drug group improved 48% from baseline while the drug only group improved 39% from baseline. This design was similar to Isomeri study, but as both are reported only as abstracts, it is difficult to fully evaluate mean scores and analyses methods. This is the third exercise/drug intervention study in FM indicating that both modalities are used in clinical practice and worthy of scientific evaluation. | | | | | | |
| 45) Valkeinen et al. [73] | RCT  26 females  Age 55-65 | | 2 groups:   1. Progressive Exercise 2. Control   PL – 21 weeks | Duration not disclosed  2/week  40% 1-RM to 80% 1 RM 6-7 exercises for all muscle groups  Repetitions/sets not disclosed | 0% Total | Tender points, VAS pain,  Cross sectional area of quads on MRI (CSA), EMG for neuronal recruitment, serum DHEAS, free testosterone, GH and IGF-1 |
| Results/Conclusions/Comments: Hormone levels remained unchanged in both groups. Progressive strength training increased strength, CSA, and voluntary activation of the trained muscles in elderly women with FM in a similar manner as matched healthy controls. This appears to be subgroup analyses of the study published by Häkkinen 2001. This analysis is important for two reasons: it examines a high-risk under investigated group: older persons with FM; it demonstrated the progressive strenuous strength training that resulted in muscle size and strength changes did not raise IGF-1 levels, suggesting perhaps that IGF-1 must simultaneously manipulated with a combination of drug and/or longer duration or aerobic type exercise interventions to normalize. | | | | | | |
| 46) Zijlstra et al. [74] | RCT  134 (128 females, 6 males)  Age 24-64 | | Two groups:  Spa treatment, exercise combined with stretching and education  Control TAU  PL – 15 consecutive days | 180 minutes  7-8 sessions spa treatment  60 minutes – 7 sessions group exercise  60-90 minutes group education  THR 70% | 31% Spa treatment  12% Control  9% Total | SF-36, VAS general health, FIQ, Beck Depression Inventory, checklist individual strength (CIS) for fatigue and physical activity, Total Myalgic Score, 6 minute walk test |
| **Results/Conclusions/Comments:** The only significant between group difference at immediate post intervention was that fatigue was improved in the treatment group compared to the control group, as measured by the CIS. There were multiple fitness and symptom improvements within the treatment group compared to their baseline scores. Nonetheless, this study is intriguing as it tests a multimodal therapy system based in part on decreasing the stress response. However, this study is limited by selection bias and feasibility of transporting subjects to treatment site, cost and lack of long term efficacy. | | | | | | |
| 1- RM, 1 repetition maximum, AGN, Percent age – predicted maximum heart rate determined by standard equations; ASES, Arthritis Self-Efficacy Questionnaire; BDI, Beck Depression Index; CBT, cognitive behavioral therapy; CV, cardiovascular training; EMG, electromyography; FIQ, Fibromyalgia Impact Questionnaire; Flex, flexibility training; FM, fibromyalgia; GH, Growth Hormone; IGF-1, Insulin-like growth factor-1; MHAQ, Modified Health Assessment Questionnaire; MHR, maximum heart rate; MPI, Multidimensional Pain Inventory; PL, program length; RCT, Randomized control trial; SF-36, Short-form 36:SIP, Sickness Impact Profile; THR, target heart rate; VAS, Visual Analogue Scale | | | | | | |
